# Spontaneous eye movements during focused-attention mindfulness meditation

**DOI:** 10.1371/journal.pone.0210862

**Published:** 2019-01-24

**Authors:** Alessio Matiz, Cristiano Crescentini, Anastasia Fabbro, Riccardo Budai, Massimo Bergamasco, Franco Fabbro

**Affiliations:** 1 PERCRO Laboratory, Scuola Superiore “Sant’Anna”, Pisa, Italy; 2 Department of Languages and Literatures, Communication, Education and Society, University of Udine, Udine, Italy; 3 Department of Psychology, University of Rome La Sapienza, Rome, Italy; 4 Department of Medicine, University of Udine, Udine, Italy; 5 Department of Neuroscience, University-Hospital “S. Maria della Misericordia”, Udine, Italy; University of Pécs Medical School, HUNGARY

## Abstract

Oculometric measures have been proven to be useful markers of mind-wandering during visual tasks such as reading. However, little is known about ocular activity during mindfulness meditation, a mental practice naturally involving mind-wandering episodes. In order to explore this issue, we extracted closed-eyes ocular movement measurements via a covert technique (EEG recordings) from expert meditators during two repetitions of a 7-minute mindfulness meditation session, focusing on the breath, and two repetitions of a 7-minute instructed mind-wandering task. Power spectral density was estimated on both the vertical and horizontal components of eye movements. The results show a significantly smaller average amplitude of eye movements in the delta band (1–4 Hz) during mindfulness meditation than instructed mind-wandering. Moreover, participants’ meditation expertise correlated significantly with this average amplitude during both tasks, with more experienced meditators generally moving their eyes less than less experienced meditators. These findings suggest the potential use of this measure to detect mind-wandering episodes during mindfulness meditation and to assess meditation performance.

## Introduction

Mindfulness meditation practitioners are skilled to intentionally sustain their focus on present-moment experiences (thoughts, emotions, feelings) with a detached attitude toward their mental contents. After an indefinite period of time, however, their minds usually drift away from the meditation object, giving rise to spontaneous thought. Sometime during this mental state, known as mind-wandering, which also extends for an indefinite period of time, practitioners become aware that they are not focused on the meditation object (e.g., breath) and try to shift their attention back to it.

This cyclic process [1,2], between the two poles of being effectively engaged in a task and being off-task, appears to be common in all human activities [3]. It occurs to a degree that probably depends on task’s mental workload [4–6], task’s perceptual load [7] and on personal characteristics [8], and yet seemingly not on the type of task or its nature [9]. During disparate activities, the time spent in mind-wandering compared to the time occupied by effective task engagement seems to range from a 1:4 to a 1:1 ratio [8–11]. It is therefore fundamental to further understand the quality, utility and neural correlates of the mind’s duality of functioning, as well as to better detect when we are in one operational mode or the other.

The basic detection method for mind-wandering episodes during task performance utilizes thought sampling. This method is based on requesting the subject to report his / her mind-wandering whenever he / she realizes that his / her attention is off-task (self-caught mind-wandering), or intermittently interrupting the subject to ask about the content of his / her thoughts (probe-caught mind-wandering). Thought sampling methods enable the investigation of mind-wandering in terms of its frequency and content [12–14], in terms of the use or not of intentionality in its initiation and continuation [15], and in terms of its relation with individual characteristics [7,8,16–18], medical conditions [4,19] and its consequences on task performance [10,20–22]. Although mind-wandering has been linked to useful mental functions such as emotional processing [23], creative thinking [24] and autobiographical planning [12,14], it has also been related to reductions in task performance, which frequently occur during repetitive tasks [25–26].

Furthermore, neurocognitive markers of mind-wandering have been obtained through the observation of ERP and fMRI data. In this regard, it has been discovered that sensory-level cortical processing is reduced during mind-wandering episodes [27,28]. Moreover, distinct brain networks appear to be active during mind-wandering vs on-task performance, namely during rest or passive thought vs active cognitive processing, with the default mode network (DMN, including anterior and posterior cingulate cortices and medial prefrontal and parietal cortices) being particularly recruited during mind-wandering. These findings are consistent across a variety of tasks (for review, see [29]), including meditation [1].

Another neurocognitive marker of mind-wandering is represented by ocular activity. Indeed, one study that measured pupil diameter (PD) during working memory and reaction time tasks has provided further evidence for the reduction of sensory processing due to mind-wandering [30]: task-evoked responses in PD were generally observed when the task required external focus and correct responses were produced, while spontaneous PD activity before stimulus presentation was linked to encoding failures and slower responses in the cognitive tasks. Eye movements, and also fixation duration, have been used as a measure of mind-wandering in individuals engaged in reading tasks, i.e. as a measure to detect mindless reading vs effective reading [11,31–34]. In other studies using reading tasks, machine learning techniques have been combined with ocular measurements in order to predict episodes of mind-wandering [35,36].

The link between eye and mind seems obvious in a reading task, whereby mind-wandering can cause disengagement and the generation of thoughts unrelated to the written text [37]. During mindfulness meditation practice, however, the eyes are usually kept closed and, as they do not have a functional role per se, little is known so far about ocular activity during meditation. To our knowledge, the only study that has explored, indirectly, this topic is that carried out by Braboszcz *et al*. [38], although their research methods and hypotheses were more focused at discovering electroencephalographic (EEG) differences between practitioners of three different meditation traditions (Vipassana, Himalayan Yoga and Isha Shoonya) and a control group during execution of meditation and mind-wandering tasks. In this study, each group engaged in a meditative session and in an “instructed mind-wandering” (IMW) session in which participants were instructed to recall autobiographical memories. Signal components associated with eye movements were examined by Braboszcz et al. just to rule out the possibility that the observed power differences across meditation and control conditions were of non-neural origin, i.e. due to ocular movements. In none of the three groups of meditators were differences found across the two tasks in terms of EEG activity generated by closed-eye ocular movements. However, in the control group, increased activity was found in the ocular-generated EEG activity (in the gamma band) during mind-wandering relative to breath meditation.

When comparing eye movements during meditation and mind-wandering, it would be important to consider what closed-eye ocular activity might reflect in these mental activities. During mind-wandering episodes, possibly also during IMW tasks in which thoughts might be quite constrained and deliberate, there could be considerable spontaneous ocular activity, as deduced by studies that have assessed the phenomenology and contents of mind-wandering, particularly in the works of Stawarczyk and colleagues [39] and Song and Wang [40]. The first mentioned research assessed the occurrence of mind-wandering episodes in individuals engaged in an attention task and reported that visual imagery was an important phenomenological characteristic of mind-wandering. During visual imagery, elicitation of eye movements has been indeed observed in wakefulness, with both open eyes [41–46] and closed eyes [47–48], as well as in REM sleep [49]. Moreover, the study of Song and Wang [40] has reported that mind-wandering thoughts are mainly episodic, i.e. the self is often projected into imagined autobiographical situations within definite spatio-temporal frames. It is known that autobiographical thoughts involve mental imagery [50–53]. Furthermore, it is worth noting that during a resting state—a condition known to prompt mind-wandering [54]—spontaneous closed-eye ocular movements have been observed in individuals who did not even report experiences of imagery visualizations during the experiment [55].

Turning back to meditation, it also seems plausible that ocular activity occurs during periods of mind-wandering happening during the practice, as well as during more specific meditation practices involving visual imagery. In relation to this, however, it is worth keeping in mind the wide variety of practices that are encompassed by the term meditation [56], and their corresponding neurophysiological correlates [57]. Thus, referring to breath meditation, a kind of practice in which practitioners try to focus their attention on a precise point of their body such as the nostrils, it may be expected that ocular activity would be reduced compared with resting or instructed mind-wandering states [38]. This may be suggested by the reports of reduced ocular activity, expressed in terms of saccadic eye movements, during non-visual attention tasks requiring focus on information available in working memory (vs. search through long-term memory), such as in auditory vigilance or word repetition tasks [58–60].

The current study aims to further investigate ocular movements activity during focused attention breath mindfulness meditation (FAM) in a group of expert meditators and compare this with ocular movements activity recorded during an instructed mind-wandering task (IMW). This latter task may elicit quite constrained and deliberate thoughts and may thus not entirely capture the spontaneous nature of mind-wandering [61]; nonetheless, it could be particularly effective in preventing expert meditation practitioners from engaging in their usual meditative practice when at rest, in particular when sitting in the meditative posture [38,62–64]. More specifically, in the current study, we analyzed eye movement data while expert mindfulness meditation practitioners were asked to either perform FAM or IMW. Based on the difference in ocular activity during FAM vs. IMW for meditation-naive subjects [38], and on the observation of reduced ocular activity during attention and working memory tasks [58–60], we expected to find higher ocular activity during IMW than FAM.

## Materials and methods

### Participants

Thirty-two voluntary participants (19 female, 13 male, age range 22–64 years, mean = 43.66, SD = 12.16) took part in the study. Sample size was based on similar EEG studies on meditation (for a review, see [65]). All participants had attended an 8-week Mindfulness-Oriented Meditation (MOM) training course within the last 5 years and were recruited through email advertisements and personal invitations sent out to all former MOM trainees. MOM training is based on the Mindfulness-Based Stress Reduction program [66] and teaches participants how to practice mindfulness of breathing, bodily phenomena and thoughts [67–72]. Specifically, in relation to the eyes, this training instructs participants to practice meditation with the eyes closed.

The group of participants had on average more than two years of experience with mindfulness meditation (mean = 2.31, SD = 1.60, in years) and self-reported to have meditated 102.59 (SD = 67.60) minutes per week in the 6 months before the recordings. During the time since their initial exposure to mindfulness meditation during the MOM training, participants practiced meditation sessions, of roughly the same duration, including the three MOM meditation exercises (mindfulness of breathing, body scan, mindfulness of thoughts).

Participants gave written, informed consent to participate in the research. The study was performed in accordance with the 1964 Declaration of Helsinki and its procedures were approved by the Ethics Committee of Azienda Ospedaliera Universitaria “Santa Maria della Misericordia” in Udine.

### Stimuli and experimental procedure

Two stimuli of equal duration (7 minutes) were used. One consisted of the practice of FAM (mindfulness of breathing): participants were requested to bring their moment-to-moment attention to the sensations generated by the air going in and out of the nose (“ānāpānasati” in Pali language) [66,73,74]. Participants were requested to bring their attention back to the nose whenever they realized that their concentration had dwindled. The second experimental stimulus consisted of an instructed mind-wandering (IMW) task, whereby subjects were requested to remember or imagine one or more events of their past or future in which they, or another person, were the protagonist.

During each recording session, data was acquired simultaneously from two subjects at a time, because a further aim of the study—to be reported separately in another research article—was to investigate the potential difference between practicing meditation in pairs vs alone. For this reason, both stimuli (FAM and IMW) were undertaken twice: participants sat side by side in the same room (a condition called CPL) and then sat separately in two different rooms (a condition called SEP). Both stimuli were undertaken by the two members of a couple in the same order: the two participants simultaneously performed FAM and then simultaneously performed IMW. Thus, each participant carried out two FAM and two IMW tasks. In order to control for the effect of procedural ordering, the presentation of stimuli in the two settings was different couple by couple, counterbalanced by setting (CPL, SEP) and task (FAM, IMW).

The space used for the experiment was dimly lit and divided into two separate rooms. Data from the members of each couple were acquired both when they were sitting side by side in the same room and when sitting separately in two different rooms. Participants wore ear caps (Shape: cylinder; Material: PVC; Noise Reduction Rating: 29 dB) and sat on their meditation pillow. During data acquisition, they were requested to sit still and in silence with eyes closed. After the task was completed, they were permitted to open their eyes, relax and move for about 2 minutes. When passing from one condition to another (from CPL to SEP or vice versa), one member of the couple moved out of or into the room where the other member was sitting. Instructions about the tasks were provided in written form to each participant, so that neither participant could see the instructions fed to the other.

The 16 recording sessions were held on 16 separate days over a 7-month period. Each recording session lasted about 2.5 hours.

### Data acquisition

We recorded data using a BrainAmp MR amplifier (Brainproducts, Munich, Germany). Subjects wore a 31-electrode BrainCap cap for the electroencephalogram (EEG) (10/20 system, with additional electrodes from the 10/10 system). They had one additional electrode placed above the right outer canthus for the electro-oculogram (EOG) and two electrodes on each shoulder for the electro-cardiogram (ECG). A reference electrode was located in the triangle between FP1, FP2 and Fz, on a midline, 2cm from Fz. The sampling rate was 250Hz and impedances were kept below 10KΩ.

**Fig 1:**
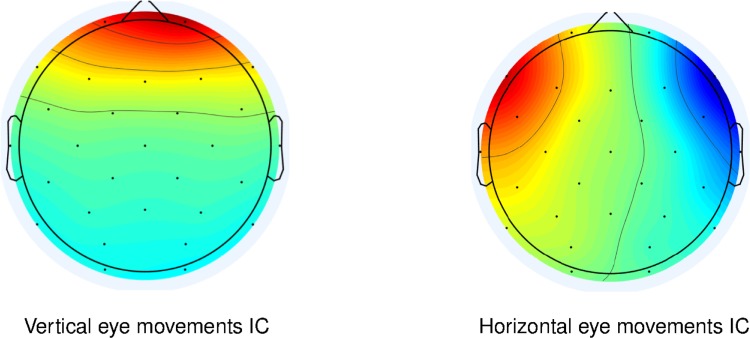
Eye movement-related Independent Components' scalp maps. The scalp maps of two sample Independent Components (ICs) extracted from EEG data, isolating activity generated by vertical (left) and horizontal (right) eye movements.

**Fig 2:**
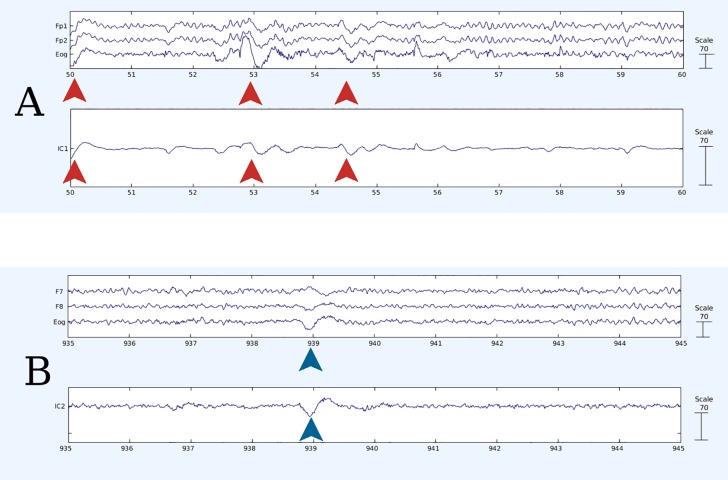
Example of ocular activity extracted through ICA algorithm. A. Vertical eye movements (some of which are marked by red arrows) are recorded on EOG channel, influence activity of Fp1, Fp2 fronto-polar electrodes (top panel) and are isolated into an Independent Component (bottom panel). B. Horizontal eye movements (one of which is marked by blue arrows) are recorded on EOG channel, influence activity of F7, F8 fronto-lateral electrodes (top panel) and are isolated into an Independent Component (bottom panel). In all plots: time is depicted on the abscissa in seconds and voltage on the ordinate in microVolts.

**Fig 3:**
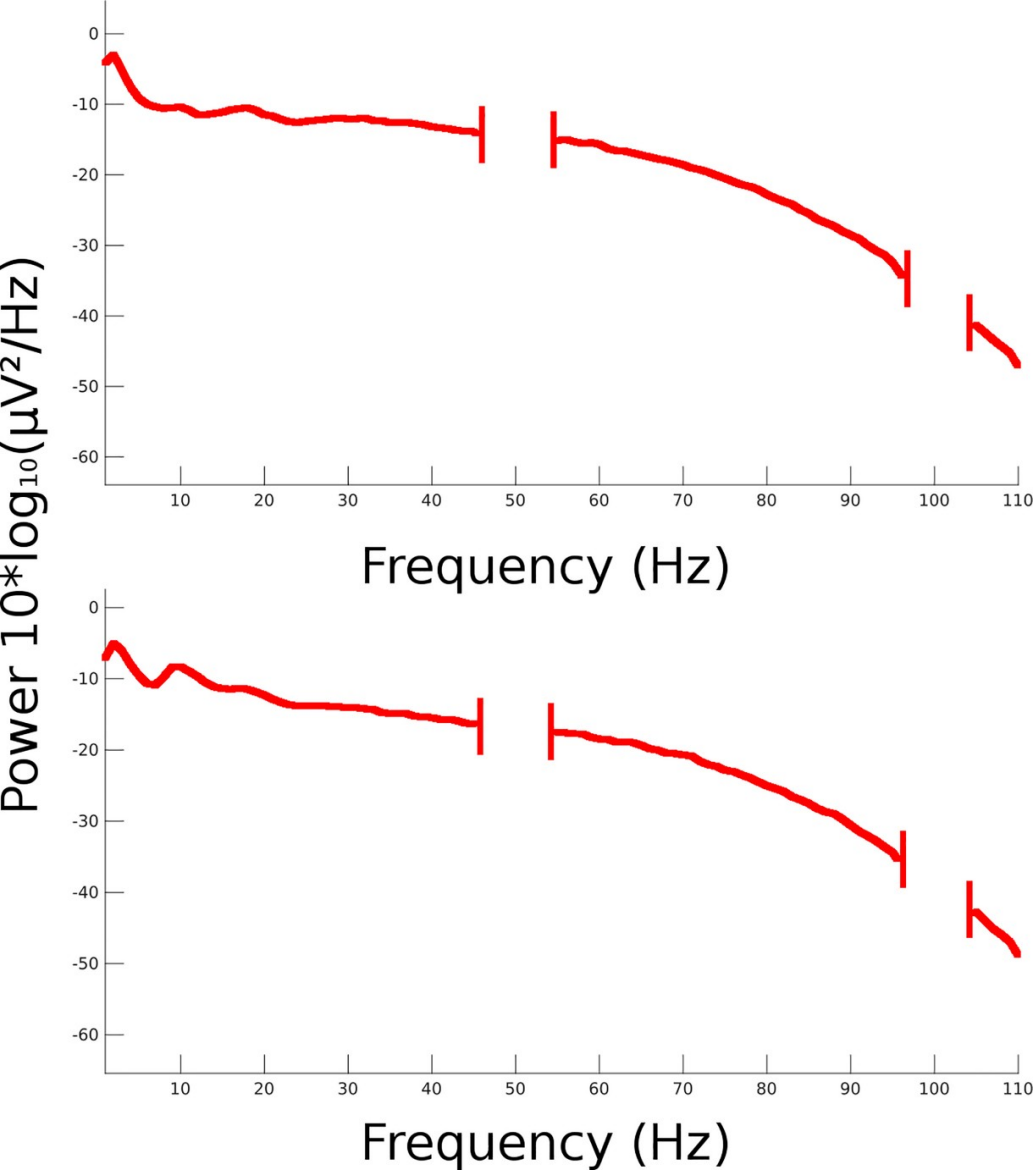
Power spectra of ocular activity. Power spectra of vertical (top plot) and horizontal (bottom plot) Independent Components of ocular activity, for a sample participant. The areas around 50 and 100 Hz have been removed from the plots and have been omitted from the analysis due to line noise harmonics.

From EEG signals, we extracted the components that do not originate from neural sources and that are due to eye movements [75,76] (Figs 1,2 and 3) through the use of an Independent Component Analysis (ICA) algorithm [77]. One-channel EOG allows the detection of combined vertical and horizontal movements and is typically used for artifact detection in EEG (e.g. [78,79]), rather than in eye-movement research. As in the Braboszcz and colleagues’ study [38], we therefore derived ocular data from EEG signals instead of using a standard multiple-electrode EOG montage to monitor ocular data. In so doing, however, we minimized participants’ awareness that their ocular movements were assessed. As oculomotor control is only partially automatic [80], we could thus rule out any major influence of voluntary control of participants’ eye movements.

### Software

Data was acquired with a Brain Vision Recorder (Brain Products GmbH, Munich, Germany) and then imported and processed with EEGLAB v.13.6.5b [81], an open source software environment running on Matlab 8.5.0 (R2015a) (MathWorks Inc, Natick, MA) under a Linux operating system (Ubuntu 16.04). The EEGLAB plug-in MARA [82,83] was used to help identify ocular activity within the EEG data.

Statistical analysis was performed using the free software environment R [84], including the following R packages: ezAnova [85] and lme4 [86] for analysis of variance, Phia [87] for post hoc tests, ggplot2 [88] for data plotting and DescTools [89] for effect sizes. Power analyses were conducted using the program G*Power 3.1 [90,91].

### Data processing and analyses

Each dataset was visually inspected and all clearly faulty channels removed (1 channel was removed from 10 subjects, 4 channels were removed from 1 subject, all others were clean). Data was band-pass filtered with a FIR filter (lower edge: 0.9 Hz, higher edge: 110 Hz) and notch-filtered to remove the line noise, before being recalculated to an average reference. In a second visual inspection of the datasets, any obvious artifactual sections caused by large muscular activity were removed. Channel data was then decomposed into maximally Independent Component (IC) processes [77] using an extended Infomax ICA algorithm [92]. An additional visual inspection was performed on the signals generated by this data transformation in order to detect more easily and eventually remove further artifactual sections that were not clearly discernible from the channel data. As a result, sections that were removed in the course of these 2 distinct manual cleaning operations, in addition to the removal of the first and last 3 seconds of each task recording, accounted for 31.4 ± 9.7 seconds for each participant, on average. Using this new data, ICA was then reapplied to obtain the conclusive ICs, among which we identified, when present, the IC for vertical eye movements (VEM) and the IC for horizontal eye movements (HEM) (Figs 1, 2 and 3).

Welch Method of Power Spectrum Estimation [93] was applied to 1 second epochs, tapered by Hanning window and no overlap, of each ocular IC. The spectrogram was then divided and averaged into the five characteristic EEG frequency bands, namely delta (1–4 Hz), theta (4–8 Hz), alpha (8–12 Hz), beta (12–25 Hz) and gamma (25–110 Hz).

Power data was log-transformed in order to extract the intra-group normality of distribution. Statistical analyses were carried out on the power of VEM and HEM ICs independently, with two repeated measures ANOVAs on the factors of Task (FAM, IMW), Condition (CPL, SEP) and Band (delta, theta, alpha, beta, gamma). In addition to these ANOVAs, a Pearson correlation test was conducted for each task between an index of meditation expertise of participants and the average power in the delta band (where we consistently found significant differences across tasks) associated with the ocular ICs during each task’s performance. For the meditation expertise index, we calculated the product between amount of weekly meditation practice (in minutes) and time spent (in years) since attendance of the MOM course for each participant. Correlation analyses were carried out separately for VEM and HEM activity.

For reasons of completeness, we also analyzed EOG data according to the same procedure described for EEG IC data. Therefore, we performed manual data cleaning, removing on average 31.7 ± 13.8 seconds of data per subject (1.9% of entire recording), power spectrum estimation and ANOVAs on average spectral power across tasks, conditions and bands.

For all statistical tests the significance threshold of p < .05 was adopted. For the ANOVAs, the assumptions of homogeneity of variance between groups of data was tested and, in case of violations of sphericity, the p-value was corrected with the Greenhouse–Geisser (p[GG]) estimate of sphericity. All post-hoc pairwise contrasts were performed using the Holm-Bonferroni procedure [94]. Generalized and partial eta squared (η_G_^2^, η_P_^2^) were adopted as measures of effect size [95,96]. In order to determine the minimum effect sizes to which the tests employed were sufficiently sensitive (Minimum Detectable Effect, MDE) [97], power analyses were performed based on current sample size, a power of 0.80, and an α level of 0.05. This power analysis revealed (see Results for details) that the experiment was generally sensitive enough to detect the differences of interest.

## Results

We identified a VEM IC for 29 subjects and a HEM IC for 23 subjects. Descriptive statistics for the power in band of these ICs are provided in Table 1.

**Table 1:** Descriptive statistics. The log-transformed power in band during the two tasks (FAM = Focused Attention Meditation on the breath; IMW = Instructed Mind-Wandering) for the Vertical Eye Movement (VEM) and Horizontal Eye Movement (HEM) Independent Components, and for EOG (electrooculogram) data. Values are expressed as mean ± standard deviation.

		VEM (N = 29)		HEM (N = 23)		EOG (N = 27)	
		Task		Task		Task	
		FAM	IMW	FAM	IMW	FAM	IMW
Band	delta (1–4 Hz)	-1.32 ± 0.56	-1.05 ± 0.59	-1.38 ± 0.35	-1.30 ± 0.38	0.48 ± 0.43	0.63 ± 0.47
	theta (4–8 Hz)	-1.71 ± 0.41	-1.57 ± 0.43	-1.70 ± 0.31	-1.67 ± 0.32	0.16 ± 0.31	0.21 ± 0.33
	alpha (8–12 Hz)	-2.04 ± 0.25	-2.01 ± 0.26	-1.90 ± 0.31	-1.90 ± 0.31	0.20 ± 0.44	0.21 ± 0.43
	beta (12–25 Hz)	-2.38 ± 0.25	-2.35 ± 0.24	-2.20 ± 0.28	-2.21 ± 0.29	-0.49 ± 0.30	-0.48 ± 0.31
	gamma (25–110 Hz)	-3.00 ± 0.27	-2.91 ± 0.27	-2.82 ± 0.25	-2.82 ± 0.24	-1.39 ± 0.21	-1.32 ± 0.22

**Fig 4:**
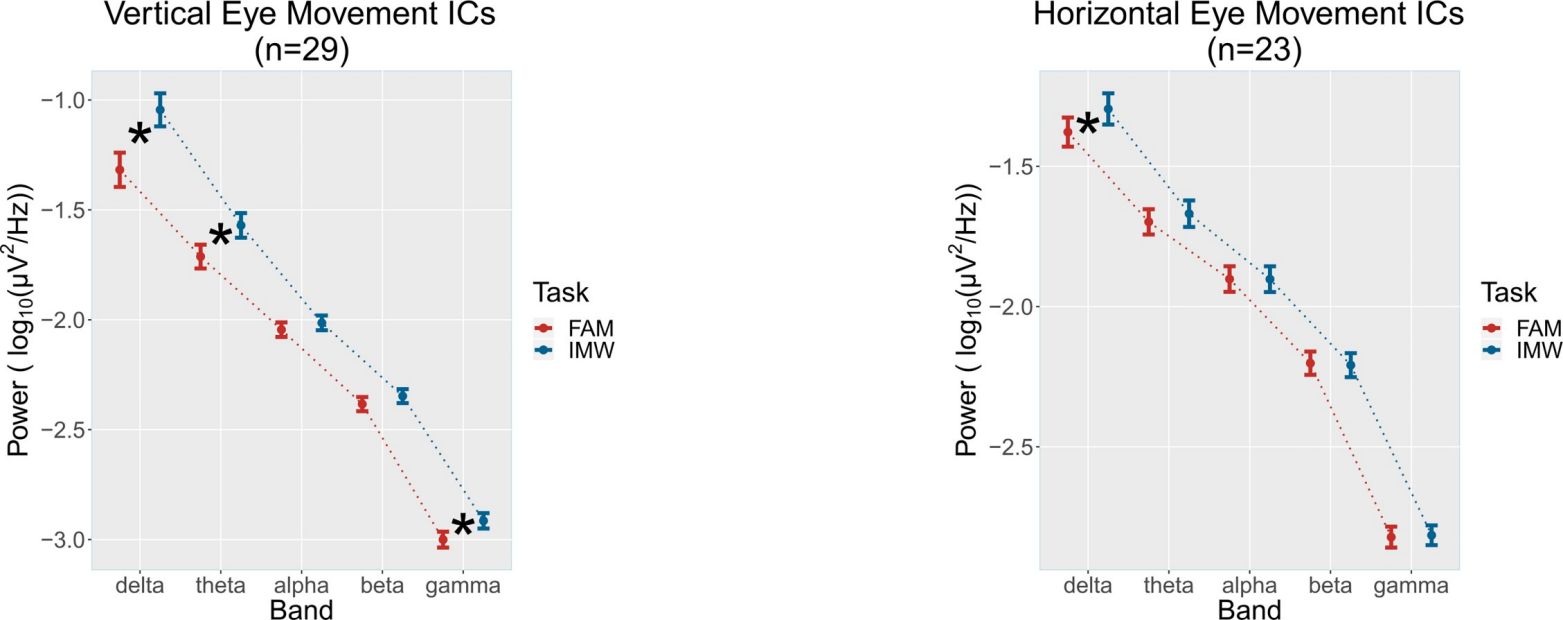
Power in band of eye movements ICs during the two tasks. Power in band relative to the two tasks (FAM = Focused Attention Meditation on the breath; IMW = Instructed Mind-Wandering) for the ICs that record activity of eye movements in the vertical (left panel) and horizontal axis (right panel). Circles represent average power across subjects, with vertical error bars for standard error of the mean. * indicates a significant difference between means for the corresponding band.

For the ANOVA on the VEM ICs, a main effect of Task (F (1, 28) = 16.97, p < .001, η_G_^2^ = .022, η_P_^2^ = .377, MDE η_P_^2^ = .244), with greater ocular activity during IMW vs FAM, was found together with a main effect of Band (F (4, 112) = 198.25, p[GG] < .001, η_G_^2^ = .725, η_P_^2^ = .876, MDE η_P_^2^ = .300) and an interaction effect between Task and Band (F (4, 112) = 15.55, p[GG] < .001, η_G_^2^ = .015, η_P_^2^ = .357, MDE η_P_^2^ = .300). For this interaction, post-hoc tests showed that the difference between FAM and IMW held in delta (p < .001), theta (p < .001) and gamma (p < .05) bands, with the greatest difference in terms of average power and probability in the delta band (Fig 4, left panel). No other effects were significant (all F < 2.62, p > .12, η_G_^2^ < .001). For the delta band filtered VEM ICs, a count of eye movements was also executed. From visual inspection of the VEM ICs data, we arbitrarily defined an eye movement when the amplitude of the signal was greater than 3 standard deviations from the signal mean. The analysis on this measure of eye movement confirmed the significant difference between the two tasks (IMW > FAM; IMW: mean = 57.8, SD = 29.5, FAM: mean = 45.5, SD = 24.1; t(28) = -3.41, p = .002, d = .632, MDE d = .539).

For the ANOVA on the HEM ICs, the main effect of Band (F (4, 88) = 261.40, p[GG] < .001, η_G_^2^ = .733, η_P_^2^ = .922, MDE η_P_^2^ = .354) and the interaction between Task and Band (F (4, 88) = 3.55, p[GG] = .04, η_G_^2^ = .003, η_P_^2^ = .139, MDE η_P_^2^ = .354) were found. Post hoc tests for this interaction showed that there was a significant difference in average power between FAM and IMW tasks (IMW > FAM) only in the delta band (p = .002) (Fig 4, right panel). No other effects were significant (all F < 1.96, p > .17, η_G_^2^ < .001, η_P_^2^ < .082).

**Fig 5:**
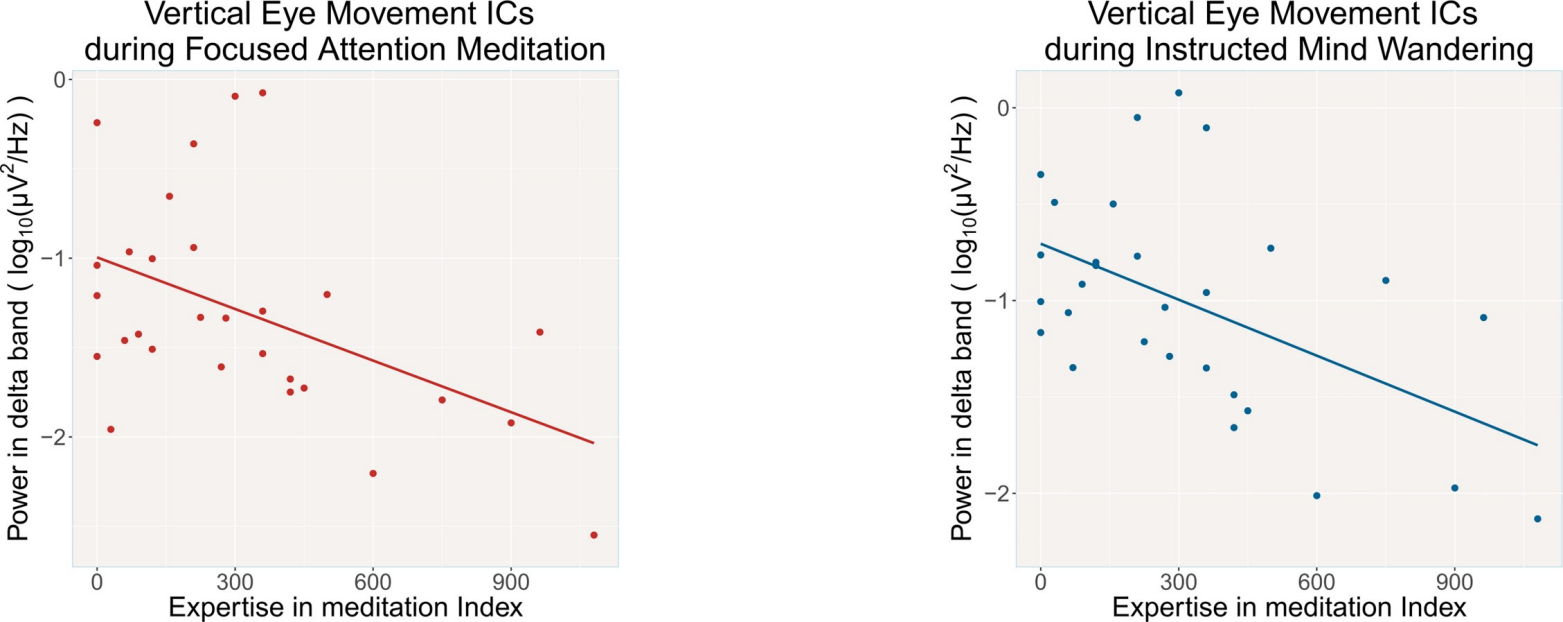
Participants' Vertical Eye Movement ICs delta power as a function of their expertise in meditation. Power in delta band (1–4 Hz) of individuals' Vertical Eye Movement Independent Component during the two tasks (FAM = Focused Attention Meditation on the breath; IMW = Instructed Mind-Wandering) as a function of individuals’ meditation expertise. Expertise in meditation Index was calculated for each subject as the product between amount of weekly meditation practice (in minutes) and time spent since attendance of the Mindfulness-Oriented Meditation course (in years).

Looking at the relationship between the meditation expertise index and the average power in the delta band of ocular activity during FAM and IMW tasks (Fig 5), we found that meditation expertise was significantly negatively correlated with VEM activity during both tasks (for FAM: r = -.48, p < .01; for IMW: r = -.52, p = .004, MDE r = -.49). This result also held for average power computed across all bands. By contrast, the expertise index was not related to HEM activity (for FAM: r = -.25, p = .25; for IMW: r = -.10, p = .64). Moreover, for both VEM and HEM activity the age of participants, which is a critical factor for eye movements metrics [98,99], was not related to average power in the delta band in either of the two tasks (all p > .05).

Finally, for EOG data, we analyzed data from 27 subjects: 5 were excluded from analysis because more than 25% of one of the their four task recordings contained artifactual EOG data. The results showed the same pattern as that for EEG data with significant main effects of Task (F (1, 26) = 15.05, p < .001, η_G_^2^ = .007, η_P_^2^ = .367, MDE η_P_^2^ = .238) and Band (F (4, 104) = 237.54, p[GG] < .001, η_G_^2^ = .786, η_P_^2^ = .901, MDE η_P_^2^ = .316), as well as significant interaction between Task and Band (F (4, 104) = 12.61, p[GG] < .001, η_G_^2^ = .006, η_P_^2^ = .327, MDE η_P_^2^ = .316). Again, average power was greater during IMW than FAM and this difference was significant in the gamma (p = .007), theta (p = .03) and delta band (p < .001). In this band, average power was significantly correlated with the meditation expertise index, both for FAM (r = −.43, p = .03, MDE r = -.51) and IMW tasks (r = −.58, p = .001, MDE r = -.51), but not with the age factor (for both tasks p > .24).

## Discussion

The aim of the present study was to assess differences in the level of spontaneous eye movement activity during a focused-attention mindfulness meditation task (FAM), with focus on the breath, and an instructed form of mind-wandering (IMW), requiring participants to remember episodes of their past or imagine events of the future. Results from analyses focused on two 7-minute executions of FAM and IMW showed significant differences between the average power associated with eye movements during the two tasks. More specifically, there was an increased level of ocular activity during IMW relative to FAM. This effect emerged for both the vertical and horizontal component of eye movements and was especially evident in the power recorded at lower frequencies (delta band, 1–4 Hz).

A previous study examining eye movements during mental time traveling, a mental state similar to IMW implying an imaginal journey into the past or the future [53], disclosed diagonal trajectories of ocular movements during the task. This result was held to be indicative of spatio-temporal associations that may link past events to more backwards and leftwards gazes and future events to more forward and right-oriented gazes. With regards to eye movements during meditation and mind-wandering, Braboszcz and colleagues [38] reported no differences between meditation and mind-wandering tasks in three groups of expert meditators, while a difference was found for naïve controls, in that they showed reduced vertical eye movements activity in gamma band (25-110Hz) during breath meditation vs IMW. In our study, this latter finding was confirmed and extended to low frequency ocular activity, with the important difference that in our study breath mindfulness meditation was practiced by expert meditators. The kind of meditation examined in our study, which demands focused attention on breathing sensations, is quite different from the three forms of meditation studied by Braboszcz and colleagues in their three groups of expert practitioners: in Vipassana meditation, participants moved their attention along their body, scanning each part of it; Isha Shoonya meditators payed attention to their thought process, in an attempt to consciously experience spontaneous trains of thoughts, emotions and sensations; finally, meditators practicing Himalayan Yoga, mentally repeated a mantra, with or without breath awareness. This last form of meditation was classified by Braboszcz et al. as a FAM practice, despite others have questioned this classification [57,100,101].

The present findings could be useful for assessing attention focus and meditation performance. We can suppose that, for a particular individual practicing breath mindfulness, mind-wandering phases may associate with a higher level of ocular movement activity than phases of effective focus on the breath; thus, meditative sessions performed with many mind-wandering episodes might be characterized by a higher level of ocular movement activation than meditative sessions performed with fewer mind-wandering episodes and longer periods of sustained attention on the breath. In line with this, one could also expect a generally higher ocular movements activity in less experienced meditators compared to more experienced meditators during breath meditation. In the present study, we took into account an index of participants’ meditation expertise, as done in other meditation studies [38,102–105], also regarding oculometric measures such as blink rates [106]. We found a negative relationship between mindfulness meditation expertise and the average power associated with vertical ocular activity during both FAM and IMW tasks: meditators with more experience generally tended to move their eyes less than practitioners with less experience. Since greater ocular activity may be associated with mind-wandering, the reduced ocular activity in expert practitioners during FAM could imply that they are better at controlling their mind-wandering during breath meditation.

Other studies have reported a relation between mindfulness meditation expertise and reduction of mind-wandering episodes, with mind-wandering being assessed both in terms of frequency and intensity through thought-probes and self-reports methods [107], or indexed by deactivation of the DMN in neuroimaging studies [108]. Moreover, it has recently been found that expert meditators show better attention and ocular sensorimotor control than novices during smooth pursuit and antisaccade tasks [109], a finding in line with the suggestion that attention and eye movement processes could involve functionally overlapping brain areas [110].

Nonetheless, the fact that participants’ meditation expertise correlated negatively in our study also with ocular activity during IMW could reflect a reduction of visual imagery during this task, a finding clearly in need of future investigations. Indeed, it may be that more experienced meditators in our study were more self-aware when they performed the IMW task, undertaking the task “more slowly” than individuals with less mindfulness meditation experience, paying more attention to what they were imagining, resulting in less eye movements.

In conclusion, it is also important to consider the relevance of the present results in terms of different types of eye movements, such as, for example, saccadic eye movements and eye fixations. During fixation periods the eye generally remains aligned with a target, but it does not remain motionless, exhibiting drifts (patterns with activity peak in the range of 1–20 Hz), tremors (with peak within 40-100Hz) or microsaccades (the largest kind of fixational eye movements) [111]. During saccades, the eye moves from one point of interest to another, so the amplitudes are larger than during fixations. Saccades are considered rapid eye movements [112], but it is known that the size of a saccade is directly related to its duration [113], so all the spectra we extracted in the current study from vertical and horizontal independent components showed a decreasing course, with larger amplitudes associated to lower frequencies. The results of our analyses, for the vertical components in particular, showed that the difference between the two tasks (FAM and IMW) in term of average power is particularly significant for the lower frequencies of ocular activity (1-4Hz), where the correlations with meditation expertise were also significant. Thus, we consider the slower and larger vertical components of saccades as the most reliable markers of the difference between the types of meditation and instructed mind-wandering tasks taken into account in our study. In general, the present findings appear to be in line with those reported in previous studies on the phenomenology of mind-wandering [39,40] and on the measure of ocular activity during visual imagery [41–49]: we detected abundance of eye movements during the IMW task, when subjects are likely engaged in mentally displaying themselves or others in spatio-temporal scenes about the past or the future.

### Limitations of the study

As already mentioned in the introduction, the IMW task used in our study to induce mind-wandering can only approximate this particular and complex mental state in which one enters in an indefinite and spontaneous series of thoughts. This is due to the fact that, although capturing mental time travel [114,115], thoughts in the IMW task might be fairly constrained and deliberate. Spontaneity is indeed considered as a key characteristic of mind-wandering [61]; nevertheless, some authors have experimentally remarked that mind-wandering can also be intentional [15,116]. In general, in order to further clarify the relation between mindfulness meditation and mind-wandering, future studies should consider the use of tasks tapping into more spontaneous aspects of mind-wandering.

Future research could also confirm our results using meditators together with a control group of non-meditators, as done in previous research [38], in order to further investigate the impact of mind-wandering, as indexed by ocular activity, in individuals not trained in focused attention practices. In this regard, in further studies it might be better to employ different types of focused-attention tasks, in order to reveal whether the present results obtained for breath meditation (vs. instructed mind-wandering) are somehow generalizable to other attention/meditation practices. This future research could also help to further examine the potential difference across meditation and mind-wandering tasks between vertical and horizontal eye movements, an issue still remaining largely unexplored in past research. Indeed, beside the fact that horizontal and vertical eye movements may be linked to distinct groups of premotor neurons [117] and may influence specific eye movement metrics [118], the current study suggests that the IMW task, more than the FAM task, is associated with an evident ocular activity in both the left/right and up/down directions.

## Conclusions

The present work investigated closed-eye ocular movements of expert meditators during instructed mind-wandering and during breath mindfulness meditation. Two main findings were obtained: a greater eye movements activity during instructed mind-wandering than mindfulness meditation and a negative relationship between mindfulness meditation expertise and ocular activity in both tasks. Taken together, these data suggest that further research could continue to explore the usefulness of using eye movement measurements during the practice of mindfulness meditation as a marker of mind-wandering and attention focus and, consequently, as an objective parameter of meditative performance.
